# Damage Indexing Method for Shear Critical Tubular Reinforced Concrete Structures Based on Crack Image Analysis

**DOI:** 10.3390/s19194304

**Published:** 2019-10-04

**Authors:** Yuan-Sen Yang, Chia-Hao Chang, Chiun-lin Wu

**Affiliations:** 1 Department of Civil Engineering, National Taipei University of Technology, Taipei 106, Taiwan; changchiahoa@gmail.com; 2 National Center for Research on Earthquake Engineering, Taipei 106, Taiwan; clwu@ncree.narl.org.tw

**Keywords:** image-based measurement, crack measurement, shear cracks, flexural cracks, damage index

## Abstract

Image analysis techniques have been applied to measure the displacements, strain field, and crack distribution of structures in the laboratory environment, and present strong potential for use in structural health monitoring applications. Compared with accelerometers, image analysis is good at monitoring area-based responses, such as crack patterns at critical regions of reinforced concrete (RC) structures. While the quantitative relationship between cracks and structural damage depends on many factors, cracks need to be detected and quantified in an automatic manner for further investigation into structural health monitoring. This work proposes a damage-indexing method by integrating an image-based crack measurement method and a crack quantification method. The image-based crack measurement method identifies cracks locations, opening widths, and orientations. Fractal dimension analysis gives the flexural cracks and shear cracks an overall damage index ranging between 0 and 1. According to the orientations of the cracks analyzed by image analysis, the cracks can be classified as either shear or flexural, and the overall damage index can be separated into shear and flexural damage indices. These damage indices not only quantify the damage of an RC structure, but also the contents of shear and flexural failures. While the engineering significance of the damage indices is structure dependent, when the damage indexing method is used for structural health monitoring, the damage indices safety thresholds can further be defined based on the structure type under consideration. Finally, this paper demonstrates this method by using the results of two experiments on RC tubular containment vessel structures.

## 1. Introduction

Sensing and quantifying damage plays a critical role in the process of structural health monitoring, which aims to detect structural damage and provide early warnings when a possible risk of failure is detected. Many structural health monitoring systems employ accelerometers, displacement sensors, or piezoelectric sensors located at selected locations to monitor changes in the structure’s deformation, natural frequencies, and modal shapes [1,2]. These systems then evaluate possible failure modes, damage levels, and locations. While accelerometers are typically employed for beam-column-based structures such as buildings, these are not the optimal sensors for structures whose failure modes are insensitive to the structure’s vibration characteristics. For some types of structures such as dams, tunnels, and reinforced concrete vessels, or shear-critical components such as reinforced concrete (RC) walls, the detection and evaluation of cracks is a relatively practical approach for safety assessment and monitoring.

Several structural damage indices have been proposed. Park et al. [3] proposed a damage index for a structural system according to its largest system displacement, ultimate displacement, accumulated strain energy, cyclic loading effect, and system yield force and displacement. Based on the calculated damage index, the structural system can be classified into one of the following damage levels: slight, minor, moderate, severely damaged, and collapsed. Roufaiel and Meyer [4] proposed a damage index that uses the initial stiffness, current stiffness, and failure stiffness. Powell and Allahabadi [5] proposed an index based on the current displacement, yield displacement, and ultimate displacement. These damage indices consider a structure as a single-degree-of-freedom system to simplify damage level estimations. However, in practical applications, these damage indices are difficult to use, as the stiffness and the displacement of a structure is sometimes difficult to measure for real, multiple degrees of freedom, and partially damaged structures. Detailed structural performance and safety may require advanced structural analyses based on finite element analysis tools [6,7] or structural experiments [8,9] which are specific to a certain type of structure. For the purpose of structural health monitoring, the displacements of certain locations can be monitored by pre-installed displacement devices; however, current stiffness and other structural properties are difficult to accurately measure or estimate. 

Alternatively, for easy to implement and quick structural safety assessments of reinforced concrete (RC) structures, several evaluation methods have been proposed that instead consider the surface cracks of concrete structures. The Japan Building Disaster Prevention Association (JBDPA) provides a guide based on the visible cracks in the concrete surface of beams, columns, or walls, and categorizes damage into five classes according to the maximum opening width of the cracks [10]. According to the JBDPA criterion, structures with a maximum crack width larger than 0.2 mm, 1 mm, and 2 mm are categorized as showing light damage, moderate damage, and heavy damage classes, respectively. The International Atomic Energy Agency (IAEA) uses a more conservative standard that categorizes cracks with an opening width larger than 0.2 mm and 1 mm as moderate and severe damage, respectively [11]. The bridge inspector’s reference manual, published by US Department of Transportation [12], categorizes cracks into structural cracks, flexural cracks on a tee beam, shear cracks on a slab, temperature cracks, shrinkage cracks, longitudinal cracks, etc.

For surface damage detection and evaluation, image-based measurement is an automatic and cost-efficient method in terms of hardware cost. As the aforementioned structural health monitoring or damage detection methods have different features, advantages, and limits, no single method can be used to replace another, nor can it be used as the sole means of structural health monitoring or damage detection. Image-based measurements, and their potential for damage detection, are not intended to replace any of the aforementioned methods. Instead, the image-based method aims to provide an area-based measurement method to measure or monitor cracks [13], strain fields [14,15], multi-axial displacement [16], or structural vibrations [17], where technology for conventional displacement measurements is inadequate [18]. The hardware cost may be relatively low [19], and may even employ existing surveillance cameras in the structure, thus eliminating the need to install additional cameras [20]. With recent dramatic improvements in digital image processing techniques, image analysis algorithms, accuracies, reliability, and computing speed have improved as well; thus, image measurement has a strong potential for practical structural health monitoring applications [21].

This work develops an image analysis-based damage indexing method following a previously developed image-based crack measurement method. This method is tested using two cyclic tests of RC containment vessels [22]. The vessels are shear critical with a large number of shear cracks induced by only a small displacement. A fractal dimension method [23] is modified and employed in this work to quantify the number of cracks. Based on the number of cracks, as well as their opening widths and orientations, a method for calculating damage indices is proposed. This method modifies the previous image analysis method [24], such that concrete surface crack orientations can be determined automatically. In addition, the fractal dimension crack analysis method [23] is modified so that the damage index can be separated into a shear damage index and a flexural damage index to distinguish between the different types of failure. The combination of these methods will make it possible to carry out structural health monitoring in an automatic manner in practical applications in the future. This paper further demonstrates the image measurement and damage indices calculation procedure based on the aforementioned RC containment vessel experiments.

## 2. Image Measurement of Cracks on Concrete Surfaces

Image-based monitoring and damage identification consists of two major procedures: image measurements and damage quantification. Image measurements analyze the image(s) of the measurement regions of interest and provide details, such as locations, lengths, opening widths, sliding displacements, and the orientation of the cracks. The damage evaluation procedure estimates the damage level or index of the measurement region according to the analyzed results from the image measurement.

Many image measurement algorithms and methods have been proposed to detect cracks on measurement regions, such as on concrete surfaces or pavements. These methods can be classified into two groups: (1) edge detection-based methods, and (2) displacement field-based methods. Edge detection-based methods are capable of finding cracks that appear as dark lines in an image. The cracks need to be of sufficient width to appear as dark lines, which is theoretically the width of a pixel. Edge detection methods [25,26,27,28] or machine learning methods [29,30,31,32] are typically employed to identify the locations or widths of cracks. A review of crack detection methods can be found in [33].

Alternatively, the displacement field-based method identifies cracks according to the displacement field of the measurement region, where the displacement field is analyzed by image analysis techniques [24,34]. Due to the high precision of image-based displacement field measurements, displacement field-based methods are capable of detecting thin cracks with widths of much less than one pixel. Yang et al. [34] detected cracks as thin as 0.2 pixels in photos in an outdoor experiment where images contained environmental light noise. The same image analysis technique detected thin cracks whose width was equivalent to 0.03 pixels in photos in a structural laboratory [24]. This type of method estimates the cracks’ opening widths, sliding displacements, and orientations, according to the change in the displacement field between each set of photos taken before and after cracks occurred, respectively. Thus, the first set of photos is used as a reference for the displacement field. Compared with edge detection-based methods, displacement field-based crack detection methods are suitable for thin crack detection, monitoring the early stages of crack development, or monitoring large regions where pixels are relatively coarsened. However, it should be mentioned that most edge detection-based methods used are tailored for inspection, rather than health monitoring. They are more suitable for that purpose than displacement field methods. In addition, displacement field methods tend to be more computationally expensive.

This work employs a displacement field-based method for crack measurement. However, this does not mean that edge detection-based methods cannot be applied to the damage evaluation method proposed in this work. The displacement field-based method is employed here because it is capable of detecting thin cracks that occur in the early stages of structural damage. In addition, the image measurement software, ImPro Stereo, is publicly available on the internet [35], and is further integrated with the damage evaluation computer codes developed in this work.

The displacement field-based method for crack measurement includes five main steps: camera calibration, measurement region positioning, metric rectification, displacement field analysis, and crack analysis. A detailed procedure can be found in [34,36]. This work only focuses on the analysis results as related to the follow-up damage evaluation procedure which is proposed herein.

**Figure 1:**
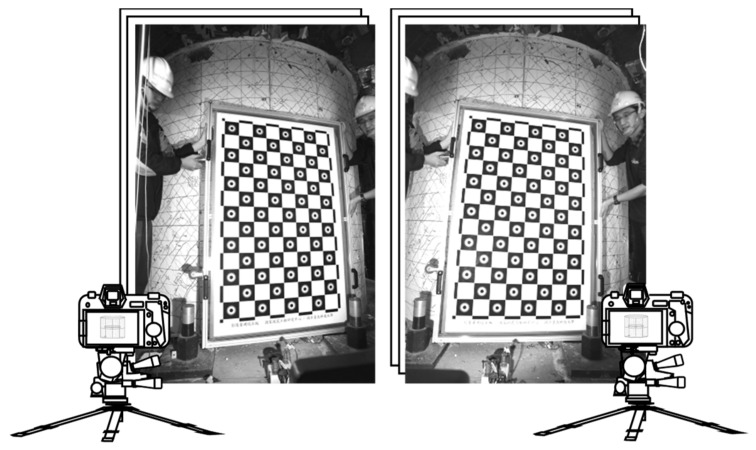
Stereo calibration of two cameras.

Camera calibration is the process of finding the intrinsic and extrinsic parameters of the camera. The intrinsic parameters are essentially its optical properties, such as the fields of view and optical distortion coefficients. The extrinsic parameters describe the camera position and its orientation. Typically, the camera calibration process is only carried out once, by taking more than 10 pairs of photos of calibration objects (such as a chessboard of known size) during camera installation (see Figure 1).

**Figure 2:**
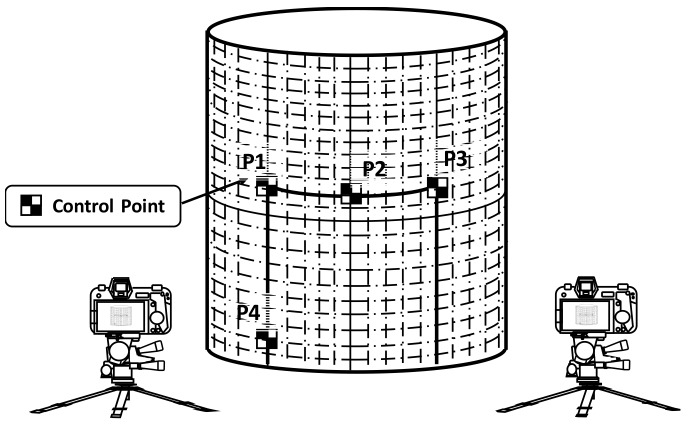
Measurement region positioning by tracking control points.

Measurement region positioning tracks the updated position of the measurement by precisely tracking the 3D positions of the control points that are used to define the measurement surface. Defining an ideal planer rectangle measurement requires at least three control points, while a cylindrical measurement region requires at least four, as shown in Figure 2. The positions of control points P1 to P4 describe the movement and deformation of the overall measurement region. Details of the process can be found in [24].

**Figure 3:**
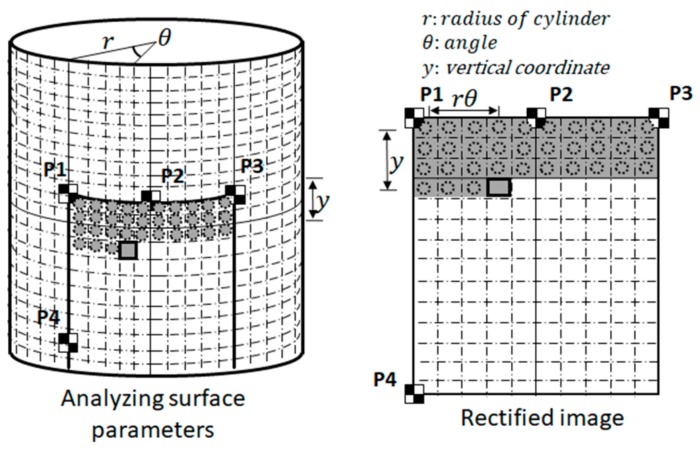
Metric rectification of the region of interest on a cylindrical structural component.

The image rectification process generates a rectangular image that represents the image pattern on the measurement region. The perspective and lens distortion effects are removed during this process. The metric rectified image can be seen as an expanded planer surface of the measurement region so that the ratio of a pixel to its physical length is constant over the entire measurement region; thus, it is essentially an image that represents the unfolded plane from the measurement region. The constant pixel-to-physical length ratio is an important property for the subsequent displacement field-based crack analysis. The rectified image is generated pixel-by-pixel, while the image intensity of each pixel is estimated by mathematically projecting a 3D point onto the surface to its image position in the photo according to the intrinsic and extrinsic parameters of the camera. Its image intensity is acquired through the numerical interpolation of neighboring pixels, as shown in Figure 3.

**Figure 4:**
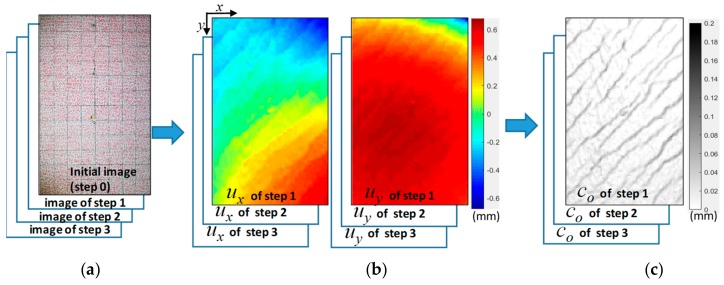
Estimating a displacement field by comparing initial and current rectified images. (**a**) Rectified images; (**b**) Displacement fields ***u*** (*u_x_* and *u_y_*); (**c**) Crack opening ($c_{o}$).

The displacement fields of the measurement region can be estimated by comparing the initial and current rectified images (see Figure 4a,b) using an object tracking method, such as template matching, digital image correlation, an enhanced correlation coefficient, or the optical flow method. Details of the process can be found in [24]. The example presented in Figure 4 was obtained from an experiment that had a measurement region of approximate dimensions of 1.4 m × 0.9 m. Each rectified image in Figure 4a is approximately 2400 × 1600 pixels. The displacement field in Figure 4b is a vector field with 90 × 60 cells, that is, each cell is represented by a sub-image with a size of 27 × 27 pixels (rounded from 2400 / 90 = 26.67). The crack opening in Figure 4c is a scalar field with the same refinement. The refinement is assigned by users, and should be tuned according to the image quality of photos when this method is being applied in practical applications. The displacement field of the rectified images is obtained by optical flow analysis [37]. The resolution of the rectified images and the refinement of the displacement and crack fields are adjusted by the user, and typically depend on the resolution and quality of the experimental photos.

Crack analysis converts a displacement field to a crack distribution. Crack analysis is suitable for thin cracks that are too thin to display as a dark line in photos, thus requiring the use of the displacement field to estimate the crack’s opening width. Each cell of the crack opening width $c_{o}$ (see Figure 4c) and crack sliding displacement $c_{s}$ of any arbitrary cell in the grid is estimated according to the displacement of its four neighboring cells. Crack sliding is the relative displacement of part A with respect to part B, i.e., parallel to the crack orientation. By using the formulation presented in [24], as shown in Equations (1)–(3), the crack distribution can be estimated by a displacement field. The crack analysis method is only suitable for brittle materials such as concrete, as it assumes that the deformation in the displacement field is mainly caused by cracks, rather than strains [34]. In addition, since the image is the appearance of the material surface, it does not represent the crack opening or sliding under beneath the surface; these are the limitations of this method. The crack distribution is a field of crack opening widths, sliding displacements, and crack orientations. It is discretized to a grid with the same grid density as the displacement. Each cell of the crack opening width $c_{o}$ and crack sliding displacement $c_{s}$ of any arbitrary cell in the grid can be calculated by Equations (1)–(3).

(1) $$\left(\begin{array}{c} c_{o}\\ c_{s} \end{array}\right)=\left(\begin{array}{cc} \cos\theta & \sin\theta \\ -\sin\theta & \cos\theta \end{array}\right)\left(u_{A}-u_{B}\right)$$

where

(2) $$u_{A}=\left\{\begin{array}{c} \frac{u_{U}\cdot \left|\cos\theta \right|+u_{L}\cdot \left|\sin\theta \right|}{\left|\cos\theta \right|+\left|\sin\theta \right|},\;\mathrm{if}\;0\leq \theta <0.5\pi \\ \frac{u_{D}\cdot \left|\cos\theta \right|+u_{L}\cdot \left|\sin\theta \right|}{\left|\cos\theta \right|+\left|\sin\theta \right|},\;\mathrm{if}\;0.5\pi \leq \theta <\pi \end{array}\right.$$

(3) $$u_{B}=\left\{\begin{array}{c} \frac{u_{D}\cdot \left|\cos\theta \right|+u_{R}\cdot \left|\sin\theta \right|}{\left|\cos\theta \right|+\left|\sin\theta \right|},\;\mathrm{if}\;0\leq \theta <0.5\pi \\ \frac{u_{U}\cdot \left|\cos\theta \right|+u_{R}\cdot \left|\sin\theta \right|}{\left|\cos\theta \right|+\left|\sin\theta \right|},\;\mathrm{if}\;0.5\pi \leq \theta <\pi \end{array}\right.$$

**Figure 5:**
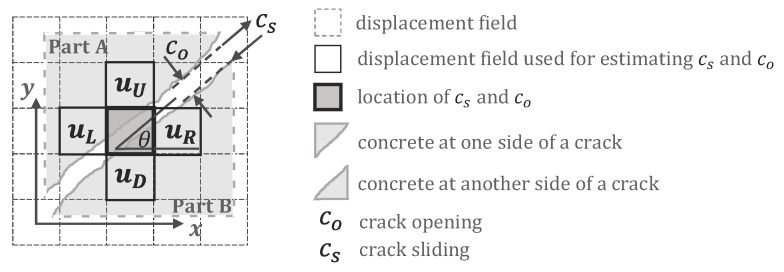
Crack opening calculation according to the analyzed displacement field.

$u_{U}$, $u_{D}$, $u_{L}$, and $u_{R}$ are the displacement vectors of the upper, lower, left, and right neighboring cells of any arbitrary cell in the displacement field, respectively (see Figure 5). The orientation of the crack of the analyzed cell is determined by iteratively testing θ within 0 and 180 degrees with a step of 15 degrees (i.e., 0, 15, 30, 45, …, 165 degrees). To be conservative, the θ which leads to the largest crack opening is selected in this method. If there is no crack on the cell, $c_{s}$ and $c_{o}$ would be very small compared with those with cracks. Small values of $c_{s}$ and $c_{o}$ are caused by either noise, image analysis errors, or relatively small strains, and are ignored in the crack analysis. Figure 4c demonstrates the discretized grid of a crack pattern estimated from its displacement in Figure 4b. It should be noted that the size scale in Figure 5 is only for demonstration. A crack is typically much thinner than the size of a cell. The cracks shown in Figure 4c are actually as thin as 0.02–0.2 mm, i.e., much thinner than the size of a cell in Figure 4b,c. In Figure 4c, the size of a cell is equivalent to a 27 × 27-pixel sub-image. While a 0.02-mm crack can be recognized by the naked eye at a close distance when inspecting damage in structural experiments, it cannot be recognized by most of the edge detection-based methods, as the crack is typically too thin to appear as a dark line in photos. In addition, human inspection is not practical for automatic structural health monitoring.

## 3. Damage Indices based on Image Analysis of Cracks

The quantification of cracks in this work is based on a Fractal Analysis of Cracks (FAC) [23]. The quantification of the total length of cracks within a measurement region can be scale dependent; the smaller the scale and the more refined the crack pattern, the more likely it is that a longer total length of cracks would be measured. A typical scale-dependent example is the measurement of a coastline, which depends on the measurement scale. This method aims to quantify the number of cracks in a more objective and scale-invariant manner, rather than directly measuring the total lengths of cracks. The FAC method adopts a fractal analysis as a benchmark method to quantify a crack by estimating its fractal dimension. While mathematically, a line is one-dimensional and a filled rectangle is two-dimensional, the dimensions of a crack distribution over a measurement region are typically a real number between 1 and 2, and do not need to be an integer. The FAC method quantifies a crack by its fractal dimension. The details of FAC can be found in [23]. 

The crack analysis method proposed in this paper modifies the FAC method. The main modifications made in this work include the following:

(1) The crack data for FAC is based on a hand-sketched crack pattern. The crack data for the modified FAC is based on an image analyzed crack pattern.
(2) The modified FAC is capable of differentiating between the damage induced by shear cracks and that of flexural cracks according to crack orientation. In this work, the crack orientation is automatically determined by finding the orientation that results in the largest crack opening.

In this work, a framework for determining the damage indices by image analysis is proposed. In this framework, the damage indices include a flexural damage index *d_F_* and a shear damage index *d_S_*. The modified FAC method to determine these damage indices is composed of seven steps. All steps have been implemented in a public software implementation developed by the authors [35].

**Figure 6:**
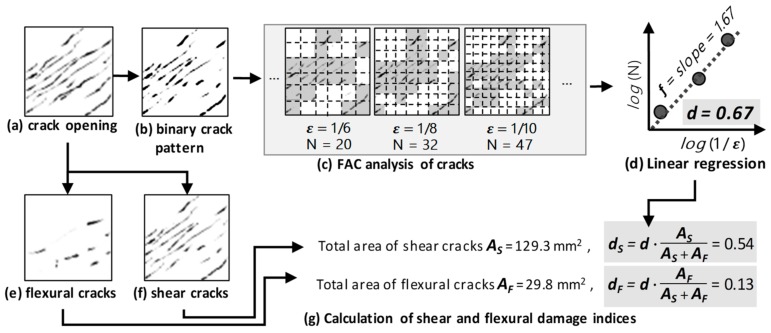
Demonstration of the proposed modified fractal analysis of cracks method. (**a**) crack opening; (**b**) binary crack pattern; (**c**) FAC analysis of crack; (**d**) linear regression; (**e**) flexural cracks; (**f**) shear cracks; (**g**) calculation of shear and flexural damage indices.

- Analyze the crack opening pattern (see Figure 6a) using the image analysis approach described above, as shown in Figure 4. In this step, the crack opening field $c_{o}$ is generated.
- Define a threshold of crack opening width, such as 0.05 mm, and convert the crack opening pattern to a binary crack pattern (see Figure 6b). The crack opening width threshold is subjective and must be determined on the basis of the actual situation. While the image analysis method in this work is capable of observing cracks as thin as 0.02 mm (see cracks shown in Figure 4c, while some of the shown cracks are as thin as thin as 0.02 mm), a threshold of 0.05 mm was chosen in this work as it is the minimum crack width in a typical crack width ruler.
- Analyze the fractal dimension by the FAC method. The FAC method is a multi-level discretization of the binary crack pattern. In each level, the crack pattern is discretized into a mesh composed of many square cells, with the number of cells that contain cracks then being counted (*N*). The width of each cell is ***ε***. At each level, *log*(1/***ε***) and *log*(*N*) can be calculated, as shown in Figure 6c. Further details of calculating the fractal dimension can be found in [26]. Note that, typically, the actual meshes in FAC analyses are more refined, and the number of discretization levels is greater (e.g., 4 levels or higher) than as shown in Figure 6.
- By applying multi-level mesh refinements (i.e., different sizes of ***ε***), *log*(*N*) versus *log*(1/***ε***) can be plotted on a 2D plot. The fractal dimension f of the crack pattern is the slope of the line found by linear regression. Since the dimension of surface crack *f* is between 1 (that is, an ideal line) and 2 (a filled area), the damage index is estimated by *f* − 1 in the FAC method. A damage index d, defined by Equation (4), is calculated, with a value between zero and one (see Figure 6d).
  (4) d=f−1
- According to the crack orientation of each crack field cell, separate the crack opening field into a shear crack opening field and a flexural crack opening field, as shown in Figure 6e,f. The crack orientation is the angle of the crack. A crack orientation of zero degree means a horizontal crack; An orientation of 45 or 135 degrees means a diagonal crack. The range of the angle is from 0 to 180 degrees. The crack orientation of each crack field cell was calculated during the crack image analysis, as shown in Figure 4. In this work, the horizontal cracks, whose orientation is between 0 to 22.5 degrees or 157.5 to 180 degrees, are classified into flexural cracks and are assigned to the flexural crack opening field, while the remaining cracks are assigned to the shear crack opening field.
- Separately calculate the total crack areas in the flexural crack opening field $A_{F}$ and the shear crack opening field $A_{S}$. Since the crack opening field represents the crack opening widths, $A_{F}$ is the summation of all values in the flexural crack opening field multiplied by the width of each cell. $A_{S}$ is calculated in the same manner.
- Calculate the flexural damage index $d_{F}$ and a shear damage index $d_{S}$ using Equations (5) and (6).
  (5) $$d_{F}=d\cdot \frac{A_{F}}{A_{S}+A_{F}}$$
  (6) $$d_{S}=d\cdot \frac{A_{S}}{A_{S}+A_{F}}$$

In most RC structures or components, crack orientation is a typical factor used to classify a crack as either flexural or shear. For RC columns or components that are subjected to bending and horizontal shear forces, horizontal cracks are typically classified as flexural, while the remaining cracks are classified as shear. This classification method is followed here. Furthermore, since the displacement field-based image analysis method provides not only the positions, opening widths, and sliding displacements of cracks, but also their orientations, it is practical to classify cracks according to their orientations. It should be noted that the classification of flexural and shear cracks by orientation is one of several classification methods, and is not necessarily applicable to all structure types. More details can be found in [10,12].

The proposed method not only integrates the previous crack image analysis [24] and FAC methods [23], but also makes some modifications. While the previous crack image analysis method requires analyzers to assign a crack orientation, the proposed method determines the crack orientation of each analyzed cell by finding the orientation that leads to the largest opening crack. While this is a conservative way to estimate crack orientation and opening width, it makes this method automatic, and does not require the orientation to be input manually. In addition, while the FAC method was originally designed for manually plotted cracks, this method uses automatically analyzed crack data for the FAC method. In the proposed method, the analyzed damage index is further separated into shear and flexural parts, providing more information on the failure mode for further safety evaluation. The integration of these methods and modifications makes it possible to carry out structural health monitoring based on crack information in practical applications.

## 4. Experiments

**Figure 7:**
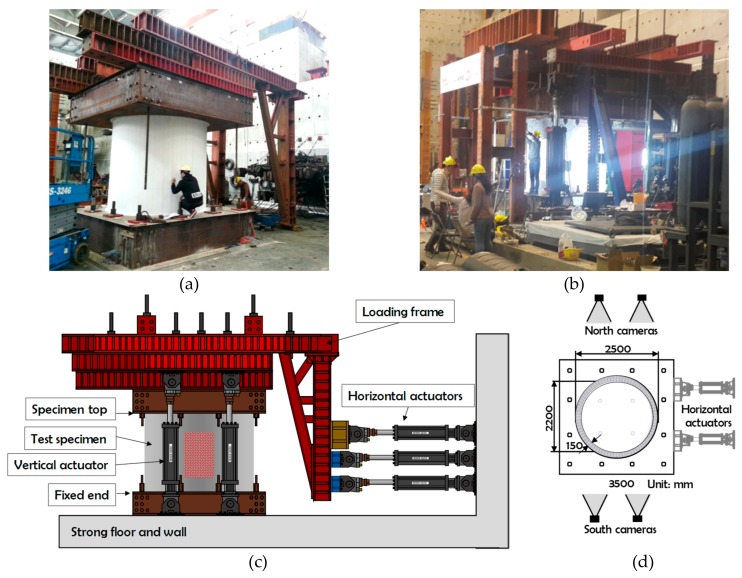
Experimental configuration and photos of both RCCV #1 and RCCV #2. (**a**) Photo of RCCV #1; (**b**) Photo of RCCV #2; (**c**) Elevation; (**d**) Plan.

The proposed image-based shear and flexural damage indices were tested using two RC structural experiments [22]. The specimens were reduced-scale RC containment vessels (RCCVs), i.e., relatively short and wide tubular structures. They are denoted as RCCV #1 (Figure 7a) and RCCV #2 (Figure 7b), respectively. The specimens were identical in terms of geometry. The specimens were subjected to a constant vertical force of 160 kN, and a cyclic horizontal displacement history imposed through hydraulic controlled actuators, as shown in Figure 7c. The outer and inner diameters were 2500 mm and 2200 mm, respectively. The height of the structures was 2250 mm. The concrete strengths of the two specimens were 37.0 and 43.4 MPa, respectively. The yields and ultimate strength of steel rebars were 379 MPa and 572 MPa, respectively. Four cameras were set up to take photos of the measurement regions, as shown in Figure 7d. The photos from the two northern cameras were used in this work. 

**Figure 8:**
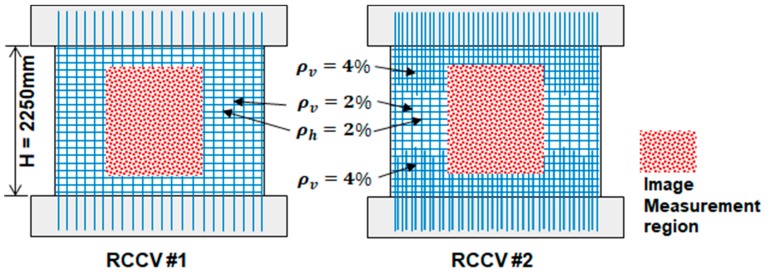
Steel rebar ratios in RCCV # 1 and RCCV # 2.

The two RCCVs had slightly different rebar designs. Four cylindrical layers of rebars were constructed in the concrete tubular structures. Each layer contained up to 90 rebars. The steel ratio of RCCV #1 was 0.02 with reinforcement extending into the top and bottom for strong interfaces between the roof, the specimen, and the foundations. RCCV #2 had gradually increasing vertical steel ratios $\rho_{v}$ near the top and bottom, as shown in Figure 8. The increased vertical steel reinforcement in RCCV #2 was designed to prevent sliding shear failure at the boundaries between the tubular structures and the top/bottom of the RC blocks, which occurred in the RCCV #1 test.

**Figure 9:**
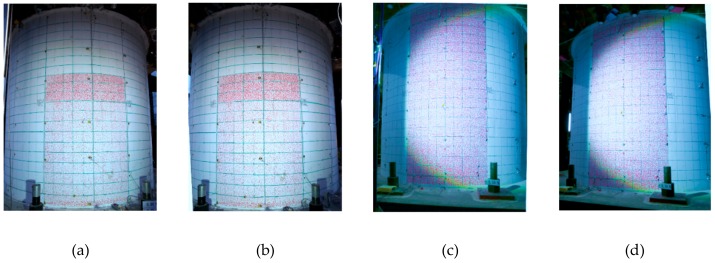
Initial photos of the two RC containment vessels (RCCV) taken from the north cameras. (**a**) RCCV #1 left photo; (**b**) RCCV #1 right photo; (**c**) RCCV #2 left photo; (**d**) RCCV #2 right photo.

Four cameras were set up in both experiments; two were positioned to the north side and two to the south, as shown in Figure 7d. Two cameras were set up for each image measurement region, because stereo image analysis was employed, as described in the previous section. The measurement regions were painted with randomly striped patterns that provided image features for the displacement fields. The lightening conditions at the top and button regions of the specimens were not as good as those in the middle regions. In addition, the middle regions had better focal conditions in the experiments. Figure 9 shows the initial photos taken by the north cameras in both experiments.

**Figure 10:**
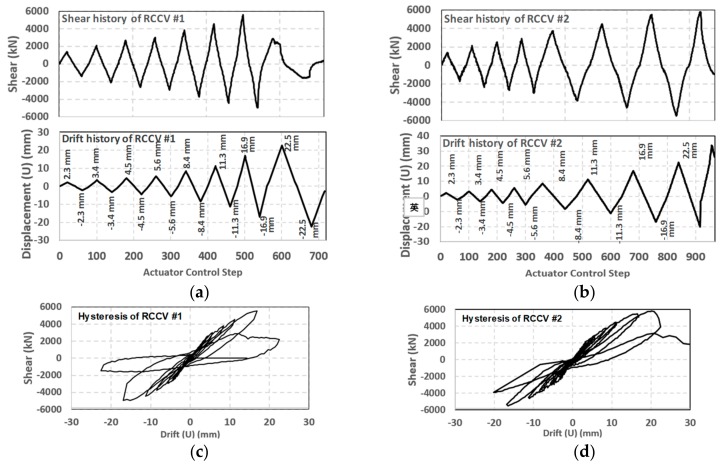
Shear/drift histories and hysteresis of RC containment vessels (RCCV) RCCV#1 and RCCV #2. (**a**) Shear and displacement history of RCCV #1; (**b**) Shear and displacement history of RCCV #2; (**c**) Hysteresis of RCCV #1; (**d**) Hysteresis of RCCV #2.

The experimental results show that the shear strength of the RCCV #2 was slightly higher than that of the RCCV #1 (see Figure 10a,b). The shear strengths of RCCV #1 and RCCV #2 were 5805 kN and 5580 kN, respectively. In addition, RCCV #1 and RCCV #2 had different ductilities. While both vessels reached their shear strengths for a displacement cycle of 16.9 mm (i.e., a drift ratio of 0.75% with respect to the specimen height of 2250 mm), RCCV #1 rapidly lost its shear strength after the 16.9 mm displacement cycle. In contract, RCCV #2 retained its shear capacity to 22.5 mm (i.e., a drift ratio of 1%), which was significantly higher because of the increased reinforcement at the top and bottom, as shown in Figure 10. The hysteresis loops of these specimens (see Figure 10c,d) show that the tangential stiffness did not significantly change until the cyclic displacements reached +/−3 mm. Details of the experimental results and explanations can be found in [22].

**Figure 11:**
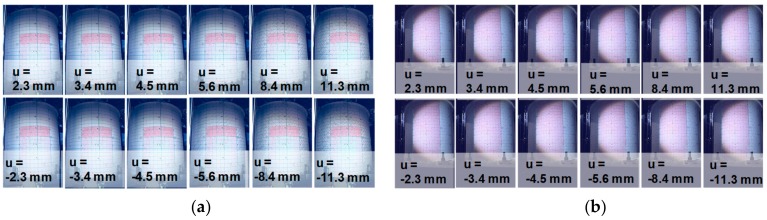
Selected experimental photos of RC containment vessels (RCCV) RCCV #1 and RCCV #2. (**a**) RCCV #1; (**b**) RCCV #2.

There were 163 and 1399 pairs of photos taken by the north cameras in the RCCV #1 and RCCV #2 experiments, respectively. Each pair of photos included a photo taken by the left camera and a photo taken by the right camera. The cameras were Canon EOS 5D Mark III with photo resolution of 3840 × 5760 pixels. Measurement regions were illuminated using a 100 W light-emitting-diode (LED). Figure 11 shows several north left camera photos of RCCV #1 and RCCV #2. The u in Figure 11 is the horizontal displacement at the top of the specimen. The displacements are so minor that the deformations are difficult to visually recognize in the figure. Since the RCCVs are shear-critical structures, a small displacement can cause significant shear failure. In addition to the experimental facilities and measurement devices, such as the load cells, the major way that we could observe the damage and the failure of the structure was to inspect the cracks on the surface. Diagonal (45-degree) shear cracks appeared on the north and south sides of the specimens, while the horizontal flexural cracks appeared at the top and bottom on the east and west sides. These cracks could be observed by human eyes only when we paused the testing, allowing people to get closer to the specimen to inspect the cracks. Details of the comparison of the manually plotted cracks and image analyzed cracks can be found in [24].

**Figure 12:**
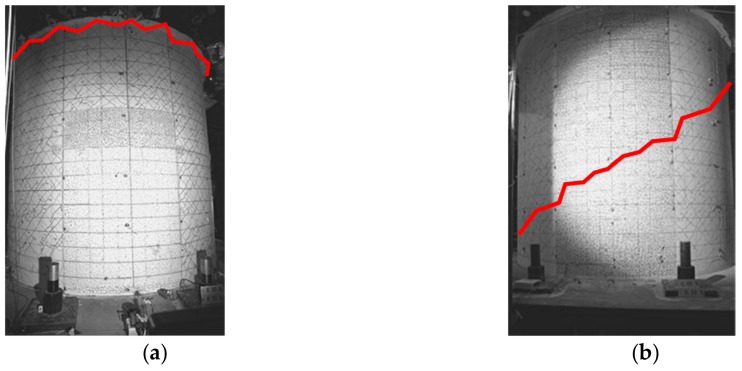
Failure modes of RC containment vessel (RCCV) RCCV#1 and RCCV #2. (**a**) Sliding shear failure of RCCV #1; (**b**) Web shear failure of RCCV #2.

While both specimens underwent shear failures, different shear failure modes were observed for each vessel. RCCV #1 had a sliding shear mode at the top of the specimen, as shown in Figure 12a. A horizontal crack occurred at the top, where the shear stiffness dramatically changes, typically inducing a stress concentration. The red lines in Figure 12 represent the locations of the cracks. Sliding shear did not occur in RCCV #2 due to the gradual change in rebar density (the steel ratio was from 2% to 4%). RCCV #2 had a web shear failure in which the major shear crack passed through the specimen at a diagonal (45-degree) angle, as shown in Figure 12b. 

**Figure 13:**
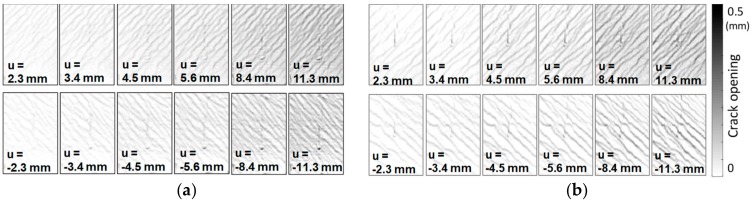
Displacement field-based crack analysis of RC containment vessel (RCCV) RCCV#1 and RCCV #2. (**a**) RCCV #1; (**b**) RCCV #2.

The crack patterns of the experimental photos, as shown in Figure 11, can be obtained by displacement field-based crack analysis. By using the displacement-based analysis, cracks as thin as 0.03 mm (approximately 0.06 pixels wide in the photos) that appeared at the very beginning of the failure could be detected. The crack patterns of the selected displacement peaks are shown in Figure 13. The crack patterns were analyzed and presented in a field discretized with a grid containing 90 × 60 cells. The size of each cell is equivalent to a sub-image with 27 × 27 pixels. In both cases, from the beginning of the tests, the cracks were distributed over almost the entire measurement region. The widths of the cracks then gradually increased from 0.03 mm (for the 2.3-mm displacement cycle) to up to 0.4 mm (for the 11.3-mm displacement cycle). 

**Figure 14:**
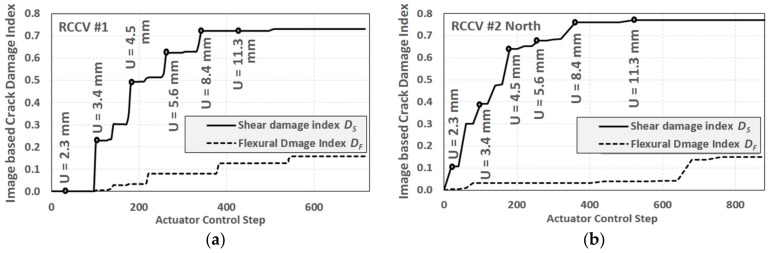
Image based damage index analysis of RC containment vessel (RCCV) RCCV#1 and RCCV #2. (**a**) RCCV #1; (**b**) RCCV #2.

The proposed crack-based damage indices are calculated on the basis of the crack pattern obtained by the displacement field-based analysis (see Figure 14). In both experiments, the shear damage increased from 0 to approximately 0.75 for the displacement cycle of 8.4 mm (i.e., drift ratio of 0.375%), and did not significantly increase after that. The shear damage indices present a warning index that is capable of capturing the early stages of shear failure. Since both RCCVs #1 and #2 are shear critical, the cracks were mostly either at 45 degrees or 135 degrees, or typical shear cracks, with relatively fewer horizontal cracks observed in the measurement regions.

**Figure 15:**
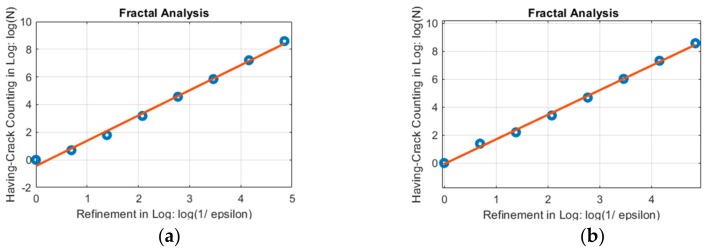
Selected fractal analysis plots of RC containment vessel (RCCV) RCCV#1 and RCCV #2. (**a**) RCCV #1; (**b**) RCCV #2.

This work examined the linear regression plots of several selected actuator control steps when analyzing the fractal dimension (as shown in Figure 6d). The plots showed that these points were very close to the line, and that the residual values were small. A selected plot of the linear regression of each specimen is shown in Figure 15. The crack pattern is a grid containing 90 × 60 cells, and is converted to different refinement of meshes with ***ε*** of 1, 2, 4, 8, 16, 32, 64, and 128 (while the most refined one is slightly more refined than the crack pattern), seven points were calculated in each of the fractal analyses.

The computing speed of the proposed method is great enough for static structural health monitoring, but still not sufficient for non-stop real-time dynamic analysis. For each step of the analysis, including image rectification, displacement field analysis, crack opening and orientation analysis, fractal analysis of cracks, and damage indices calculation, it takes about 40 seconds of computing time using a laptop equipped with an Intel i5-7300HQ 2.5 GHz processor and 32GB main memory. Sufficient computing speed may allow us to carry out automatic, non-stop crack detection and health monitoring with a sampling rate of 0.025 Hz, that is, once or twice per minute. It is still insufficient for detecting dynamic responses during a vibration event such as an earthquake, which typically requires a sampling rate of 200 Hz to 1000 Hz. To achieve non-stop dynamic analysis for structural health monitoring, this method requires not only a significant improvement in camera and computing hardware, but also further optimization of the algorithms and programming code.

## 5. Conclusions

This work proposed a damage indexing method based on crack image analysis, with the aim of indicating the early stage failure of shear critical RC structures. This method is based on a displacement field-based crack image analysis method, which is capable of detecting early stage, thin cracks on concrete surfaces. It is especially practical when displacement sensors and load cells are not applicable in real structures. Early stage, thin cracks can be detected when they are as thin as 0.03 mm, which is considerably thinner than the width of a pixel in a digital photo, and cannot be visually seen as a dark line. Based on the crack image analysis, a previously proposed fractal analysis of cracks was employed to estimate the overall damage index. According to the crack orientations, this method separates the fractal analysis damage index into a shear damage index and a flexural damage index to distinguish between the different types of failure. The software implementation method is publicly available. 

The results of two RCCV experiments were used to verify the proposed damage indexing method. Since both RCCV specimens were shear critical structures, the analyzed damage indices showed that the shear cracks dominated the major failure. The flexural crack indices were relatively low throughout the experiments. In both experiments, the shear damage indices reached a relatively high value (i.e., 0.7) at a displacement of only 8.4 mm on the top of the specimen (i.e., a drift ratio of 0.375%). Earlier damage could be detected when the displacement was only 3.4 mm (i.e., a drift ratio of 0.15%) or even earlier, while the stiffness was still unchanged. This indicates that the crack image analysis-based damage indexing method is capable of indicating early stage failure in shear critical structures. 

While this method estimates the damage indices of a structure, damage indices obtained from different types of structures are not comparable. The safety of a structure depends on many factors, including complicated design details such as the design of ties and stirrups, which are not visually observable. A non-ductile structure having a lower damage index does not mean it is safer than a ductile structure with a higher damage index. The practical health monitoring application of this method to other structures still requires sufficient experiments and investigations based upon the specific structure type.
